# The 2024 Outstanding Contributions to ISCB Award—Dr Scott Markel

**DOI:** 10.1093/bioinformatics/btae285

**Published:** 2024-06-28

**Authors:** Mallory L Wiper

**Affiliations:** The International Society for Computational Biology

Each year, ISCB presents the Outstanding Contributions to ISCB Award to recognize a society member’s contributions to ISCB through their exemplary leadership, education, and/or service. This year, the outstanding service award is being presented to Dr Scott Markel.

**Figure:**
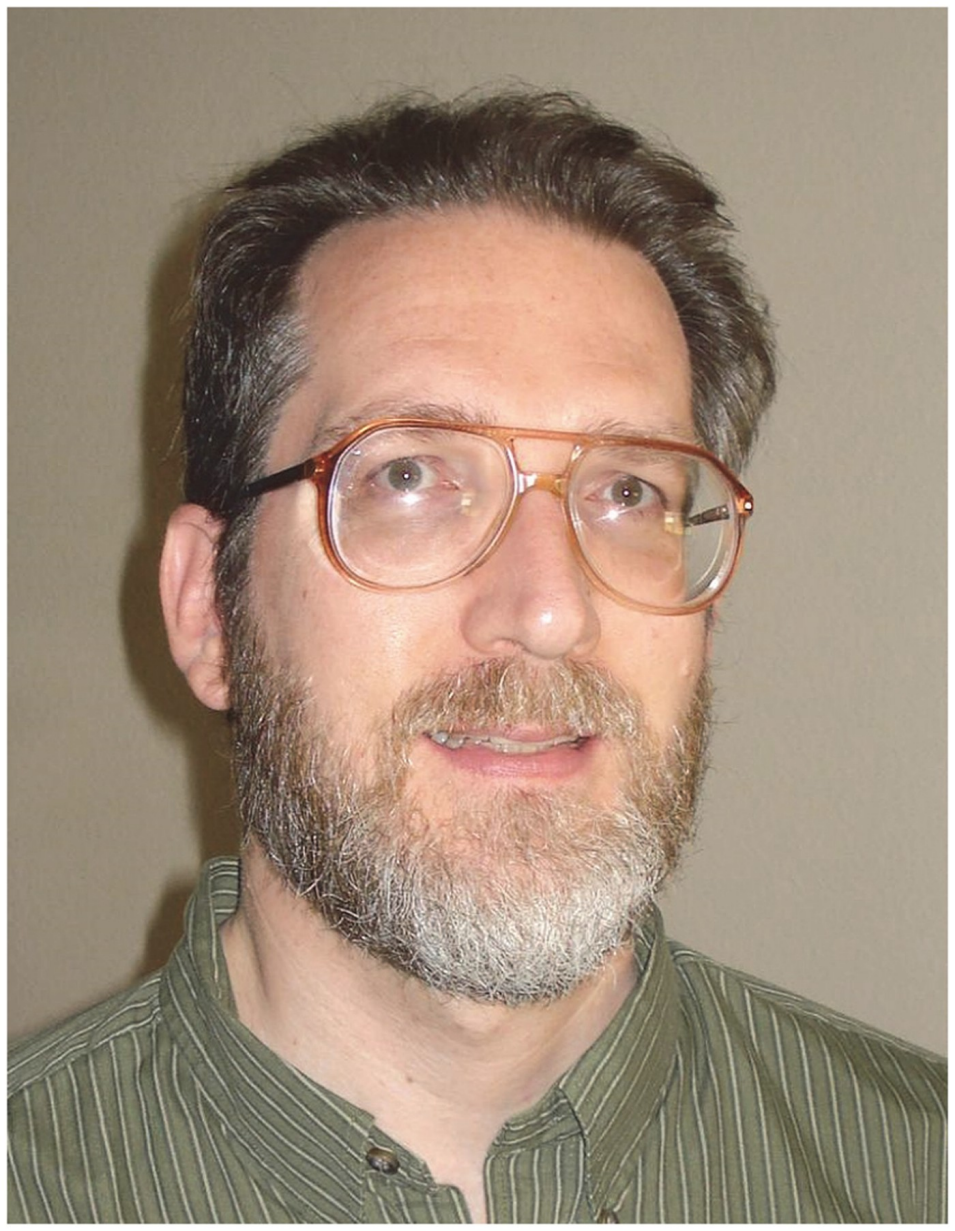


Dr Markel’s involvement with the International Society for Computational Biology began in the early 2000s when the Society was growing steadily around the Intelligent Systems for Molecular Biology (ISMB) conference. At one of the earliest ISMB conferences Markel attended, he took part in a Birds of a Feather meeting that helped to connect people in computational biology. This is when Markel met B.J. Morrison McKay, the executive director of ISCB at the time, who gave a presentation about the Society. Here is where Markel learned about the committees within the Society.

As his first foray into the organizational and administrative side of ISCB, Markel first got involved with ISCB’S Publications and Communications Committee and “things snowballed from there.” But they certainly snowballed in a good way, seeing Markel become more deeply involved in the Society’s activities as ISCB continued to grow!

While he is stated that he is less hands-on with Society activities these days, Markel was previously the Secretary for ISCB’s Board of Directors, a position that he held for a decade. In addition to his role as Secretary and his initial involvement with the Publications Committee, Markel has also been involved in the Finance Committee, Fundraising Committee, and the Nominations Committee, the latter of which, he said, gave him a unique perspective of the society. With so much involvement in so many places within the society, it is clear that Markel’s influence is not only invaluable, but widespread.

## Service opportunities with ISCB

When asked what service opportunities he had found most gratifying in his position with ISCB, Markel said that something he loved the most was being able to be part of “the magic behind the scenes of helping organize the smooth running of the Society.” The most notable service provided to the Society according to Markel, however, was heading the search committee that found the current CEO of ISCB, Diane Kovats, when McKay was stepping down.

In addition to helping find McKay’s replacement, Markel was involved in outlining the rules of Society governance so there was a strong system in place. In particular, he was involved in introducing term limits for officers and board members, and in introducing the President Elect and Past President roles, which aid with a smooth transition between presidents and helps to ensure a clear transfer of knowledge.

All in all, Markel has been a key component in bringing structure and continuity to the society’s leadership and administration, and his efforts will be appreciated for as long as the society continues!

## Searching for service opportunities

For junior scientists and trainees looking for service opportunities in the society or in the world of science in general, Markel says: “Be curious. Experience things. And don’t be afraid to share your experiences.”

He suggested three specific areas that trainees can consider when it comes to seeking out such opportunities: the technical/science side, the service side, and the educational side.

When it comes to the technical/science side of things, Markel suggests starting with things like reviewing papers and growing into bigger roles by volunteering to organize and chair special sessions at scientific meetings.

From the service perspective, the suggestion is to find a committee or Community of Special Interest (COSI) to join and see what opportunities are available within that community. Markel urges students and trainees to be willing to volunteer, take on new challenges, and experience as much as possible.

From the educational side of things, the advice from Markel is for students to think about how people helped them when they were coming into the academic world and how they might be able to do something similar to pay it forward. How did mentors help foster growth? What lessons did they share that cannot be learned in the classroom? Markel also suggests involvement in panel discussions to share experiences with things like getting published and writing grants. He also says to write articles! If there’s specialized information a student has to share, they shouldn’t shy away from doing so. Do not be afraid to put your knowledge out there!

Markel did offer another sage piece of advice for trainees looking for service opportunities: Do not commit yourself to something if you do not have the work ethic and follow-through to go along with it.

## The future of ISCB

The role of ISCB in supporting the computational biology community “will depend on where those in charge of the society lead it,” says Markel, but he sees it going in a great direction, especially since ISCB already has some very strong pillars of support for the computational biology community.

For instance, the jobs board and career center information provided on the website and at ISMB’s career fair help students see what is possible outside of academia. This is invaluable in supporting the next generation of scientists, providing exposure to opportunities they might not have known about.

The Society is also a great bridge between academia and industry, clearly connecting the two in a research area that is growing rapidly, and that Markel foresees is going to be big.

An area where Markel sees ISCB already doing great things but where he hopes to see the society continue to grow is in providing an infrastructure to support the academic community within computational biology, and sharing and spreading knowledge within this area. Namely, ISCB can continue to support and provide access to COSIs for students and trainees to find like-minded researchers and the society can also continue to strengthen its publication arm, promoting the cutting-edge research happening in the field and continuing in ISCB’s mission of spreading the science!

